# Supplementary material for: SpatialExperiment: infrastructure for spatially-resolved transcriptomics data in R using Bioconductor
Source: Bioinformatics. 2022 Apr 28;38(11):3128–31. doi: 10.1093/bioinformatics/btac299 (PMC9154247; doi:10.1093/bioinformatics/btac299)
Supplement: btac299_Supplementary_Data [file btac299_supplementary_data.zip › spatialexperiment_supplement_2022-04-22.pdf]

Supplementary Tables

Dataset name	Platform	Type	Tissue	Number of samples	Number of spots or cells	Number of features (genes)	Contains ground truth labels?	Contains image data?	Source
Visium_humanDLPFC	10x Genomics Visium	Spot-based	Human brain	1	3,639	33,538	Yes	Yes	(Maynard et al., 2021; Pardo et al., 2021)
Visium_mouseCoronal	10x Genomics Visium	Spot-based	Mouse brain	1	2,702	32,285	Yes	Yes	(10x Genomics, 2021b)
seqFISH_mouseEmbryo	seqFISH	Molecule-based	Mouse embryo	1	11,026	351	No	No	(Lohoff et al., 2021)
ST_mouseOB	Spatial Transcript-omics	Spot-based	Mouse brain	1	262	15,928	Yes	No	(Ståhl et al., 2016)
SlideSeqV2_mouseHPC	Slide-seqV2	Spot-based	Mouse brain	1	53,208	23,264	Yes	No	(Cable et al., 2021; Stickels et al., 2020)

Supplementary Table 1. Summary of example datasets provided in *SpatialExperiment* format in the *STexampleData* package. Table columns describe characteristics for each dataset, and provide the original references. For the *Visium_humanDLPFC* and *seqFISH_mouseEmbryo* datasets, the objects in the *STexampleData* package contain small subsets of the full original datasets, allowing users to easily download and load these datasets for examples and tutorials. The full datasets can be obtained from the original references.

Dataset name	Tissue	Number of samples	Targeted panel(s)	Number of spots	Number of genes
HumanBreastCancerIDC	Human invasive ductal carcinoma breast	2	–	7,785	36,601
HumanBreastCancerILC	Human invasive lobular carcinoma breast	1	–<br>*Immunology*	4,325	36,601<br>*1,056*
HumanCerebellum	Human cerebellum	1	–<br>*Neuroscience*	4,992	36,601<br>*1,186*
HumanColorectalCancer	Human invasive adenocarcinoma of the large intestine	1	–<br>*Gene signature*	3,138	36,601<br>*1,142*
HumanGlioblastoma	Human glioblastoma multiforme	1	–<br>*Pan-cancer*	3,468	36,601<br>*1,253*
HumanHeart	Human heart	1	–	4,247	36,601
HumanLymphNode	Human lymph node	1	–	4,035	36,601
HumanOvarianCancer	Human ovarian endometrial adenocarcinoma	1	–<br>*Immunology*<br>*Pan-cancer*	3,493	36,601<br>*1,056*<br>*1,253*
HumanSpinalCord	Human spinal cord	1	–<br>*Neuroscience*	2,812	36,601<br>*1,186*
MouseBrainCoronal	Mouse brain (coronal plane)	1	–	2,702	32,285
MouseBrainSagittalAnterior	Mouse brain (sagittal slice of the posterior)	2	–	5,520	32,285
MouseBrainSagittalPosterior	Mouse brain (sagittal slice of the anterior)	2	–	6,644	32,285
MouseKidneyCoronal	Mouse kidney	1	–	1,438	32,285

Supplementary Table 2. Summary of example datasets provided in *SpatialExperiment* format in the *TENxVisiumData* package. All data are spot-based, and were obtained using the 10x Genomics Visium platform (10x Genomics, 2021a). Table columns describe characteristics for each dataset. For some datasets, targeted expression panels were measured in addition to whole-transcriptome analysis; these are indicated with the name of the panel and corresponding number of genes in italics. The original datasets can be obtained from (10x Genomics, 2021c).

Supplementary Figures

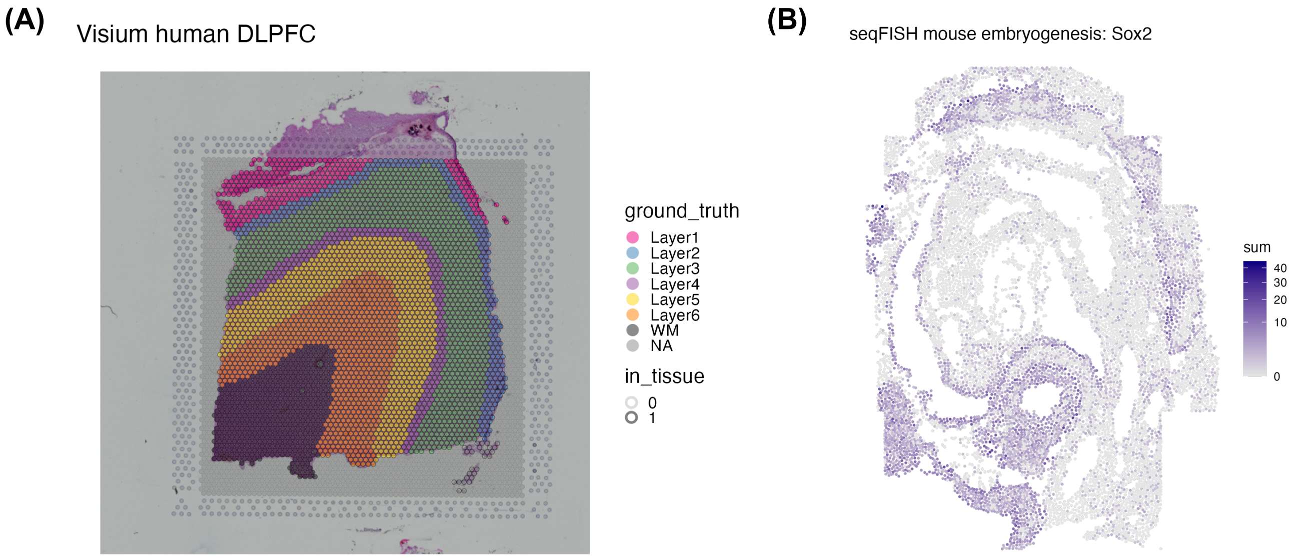

Supplementary Figure 1. (A) Example of visualization of spot-based ST data (*Visium_humanDLPFC* object from the *STexampleData* package). Image shows a histology image as background, grid of spatial coordinates (spots), highlighting for spots that overlap with tissue, and colors for ground truth cluster labels. The dataset represents a single biological sample (sample 151673) from the human brain dorsolateral prefrontal cortex (DLPFC) region (Maynard et al., 2021; Pardo et al., 2021), measured with the 10x Genomics Visium platform. The full dataset contains 12 biological samples, and is available in *SpatialExperiment* format in the *spatialLIBD* Bioconductor package (Maynard et al., 2021; Pardo et al., 2021). **(B)** Example of visualization of molecule-based ST data (*seqFISH_mouseEmbryo* object from the *STexampleData* package). Color scale shows total mRNA counts per cell for the *Sox2* gene. The dataset represents a subset of cells (embryo 1, z-slice 2) from a published dataset investigating mouse embryogenesis (Lohoff et al., 2021), generated using the seqFISH platform. Additional details on the datasets are provided in Supplementary Table 1. Figures were generated using plotting functions from the *ggspavis* package.
