# Supplementary figures and images for: SpatialExperiment: infrastructure for spatially-resolved transcriptomics data in R using Bioconductor
Source: Bioinformatics. 2022 Apr 28;38(11):3128–31. doi: 10.1093/bioinformatics/btac299 (PMC9154247; doi:10.1093/bioinformatics/btac299)

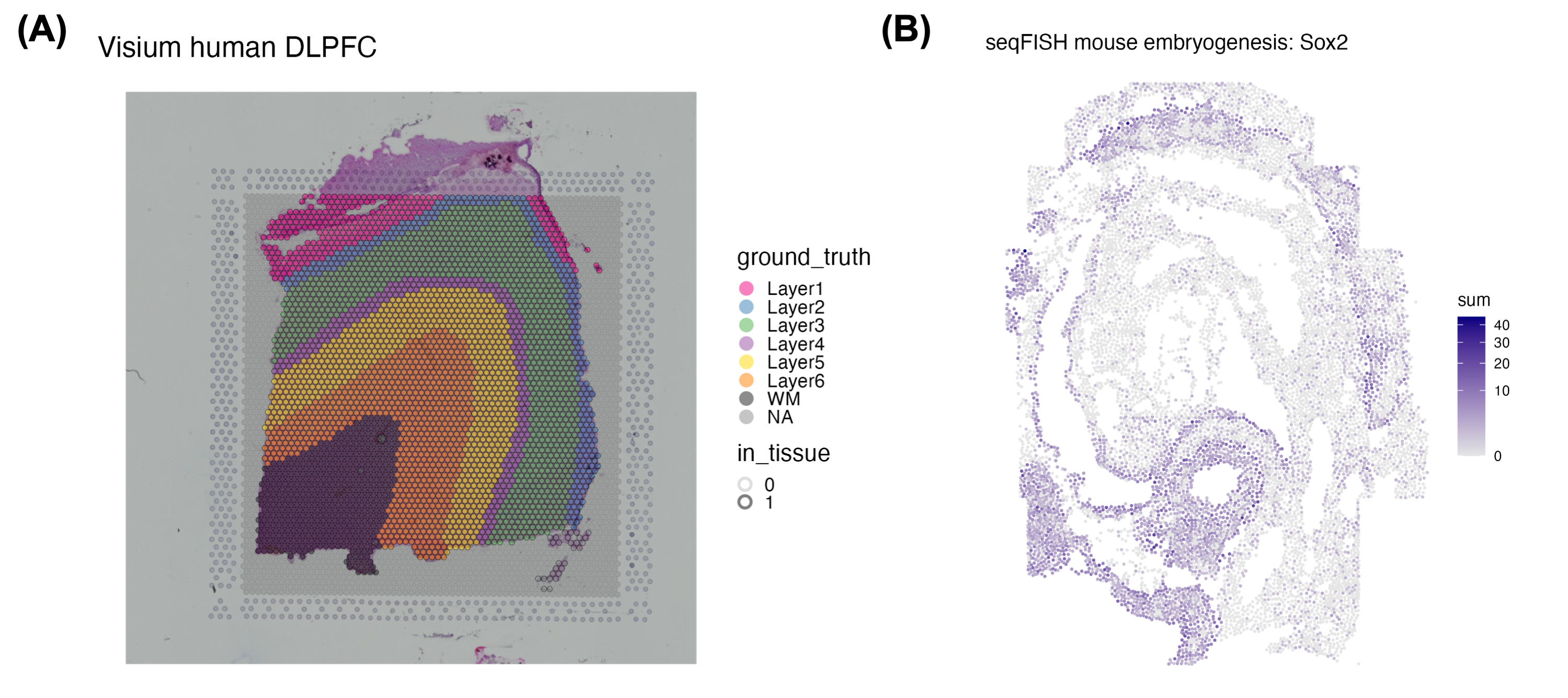

Supplement: btac299_Supplementary_Data [file btac299_supplementary_data.zip › SupplementaryFigure1_visualizations.png]
